# Spinocerebellar ataxia type 31 associated with REM sleep behavior disorder: a case report

**DOI:** 10.1186/s12883-019-1238-1

**Published:** 2019-01-11

**Authors:** Kazumasa Shindo, Tohko Sato, Hiroaki Murata, Yuta Ichinose, Takanori Hata, Yoshihisa Takiyama

**Affiliations:** Department of Neurology, University of Yamanashi, 1110 Shimokatou, Yamanashi, 409-3898 Japan

**Keywords:** REM sleep behavior disorder, Spinocerebellar ataxia type 31, Polysomonography, Cardiac ^123^I-MIBG scintigraphy, α-Synucleinopathy

## Abstract

**Background:**

Spinocerebellar ataxia type 31 (SCA 31) is a slowly progressive neurodegenerative disorder characterized by pure cerebellar ataxia. Unlike other CAG repeat diseases, sleep-related problems have not been reported in patients with SCA 31 so far.

**Case presentation:**

A 67-year-old woman was admitted to our hospital with dysarthria and gait disturbance after onset age of 62 years. Neurological examination revealed pure cerebellar ataxia. Genetic analysis detected expansion of a TGGAA repeat in the coding region of the BEAN/TK2 gene on chromosome 16p22.1, confirming the diagnosis of SCA 31. One year later, her husband noticed the patient talking loudly during sleep once or twice a week. Overnight polysomnography showed rapid eye movement sleep without atonia. Cardiac scintigraphy with iodine-123-labeled meta-iodobenzylguanidine revealed a low heart/mediastinum ratio, indicating reduced uptake, and a high washout rate.

**Conclusion:**

To our knowledge, this is the first report of a patient with SCA 31 associated with rapid eye movement sleep behavior disorder (RBD). In the future, evaluation of autonomic function, assessment of the frequency of RBD, and performance of cardiac iodine-123-labeled meta-iodobenzylguanidine scintigraphy in a larger number of SCA 31 patients could be useful to resolve important issues regarding the mechanism of RBD.

## Background

Spinocerebellar ataxia 31 (SCA 31) is a slowly progressive neurodegenerative disorder characterized by onset in adulthood and pure cerebellar ataxia with or without nystagmus and hearing impairment [1, 2]. Genetic analysis has revealed an insertion of TGGAA pentanucleotide repeats ranging in length from 2.5 to 3.8 kb between the *brain expressed, associated with Nedd4* (BEAN) and the *thymidine kinase 2* (TK2) gene and the gene located on chromosome 16p22.1 [3]. Among the various types of autosomal dominant cerebellar ataxia, SCA 31 is relatively common in the Japanese population [2], while it is rare in other East Asian countries such as Korea and China [4, 5], and extremely rare among Caucasian populations [6].

Rapid eye movement (REM) behavior disorder (RBD) is a sleep disorder characterized by repetitive episodes of talking while asleep, calling out in a loud voice, sleep walking, and falling out of bed at night, while the affected person remains soundly asleep [7, 8]. The diagnosis can be confirmed by lack of epileptic discharges on electroencephalography and by detecting REM sleep without atonia on polysomnography (PSG) [7]. Idiopathic RBD has been reported in 0.5–2% of healthy persons [8]. In addition, patients with neurodegenerative disorders, including α-synucleinopathies such as Parkinson’ s disease and dementia with Lewy body disease, frequently develop RBD during the period before and after the onset of motor symptoms [9, 10]. Among the subtypes of SCA, almost a half of patients with Machado-Joseph disease (MJD) has been reported to develop RBD [11]. However, SCA 31 has not been associated with RBD to our knowledge, and the patient described here is the first case to be reported.

## Case presentation

Written informed consent to this report was obtained from the patient. A 67-year-old Japanese woman was evaluated at the outpatient clinic of our hospital for slow progression of unsteadiness of gait/loss of balance and speech difficulty since five years earlier. She was taking two medicines (a calcium channel blocker for hypertension and a therapeutic agent for dyslipidemia) and using eye drops for glaucoma since before being hospitalized. The family history revealed that her mother had developed similar unsteadiness of gait and speech disturbance. At the initial assessment, neurological examination revealed no cognitive impairment. There was break-up of smooth pursuit eye movements, mild cerebellar dysarthria and limb ataxia, and a wide-based gait. She did not have sensory deficit nor extrapyramidal symptom. The score on the Scale for the Assessment and Rating of Ataxia was 11.5 points. The score on the Montreal Cognitive Assessment was 28/30 points. Laboratory tests, chest X-ray film, electrocardiography, and echocardiography all gave normal results. Brain magnetic imaging showed mild cerebellar atrophy. Slight cerebellar hypoperfusion was observed on radioisotope scintigraphy of cerebral blood flow. Genetic analysis revealed expansion of a TGGAA repeat insertion in the coding region of the BEAN/YK2 gene on chromosome 16p22.1, confirming the diagnosis of SCA 31.

**Fig. 1:**
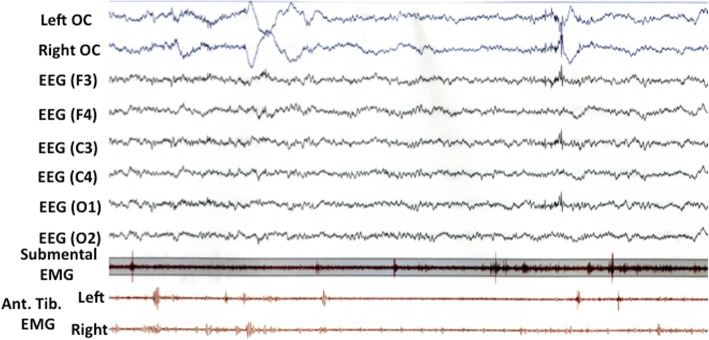
The polysomnographic finding in this case. Excessive phasic/transient muscle activity confined to chin and anterior tibialis muscles (tenth and eleventh channels) was observed during rapid eye movement sleep. OC, oculogram; EEG, electroencephalogram; EMG, electromyogram, Ant. Tib., anterior tibial muscle

**Fig. 2:**
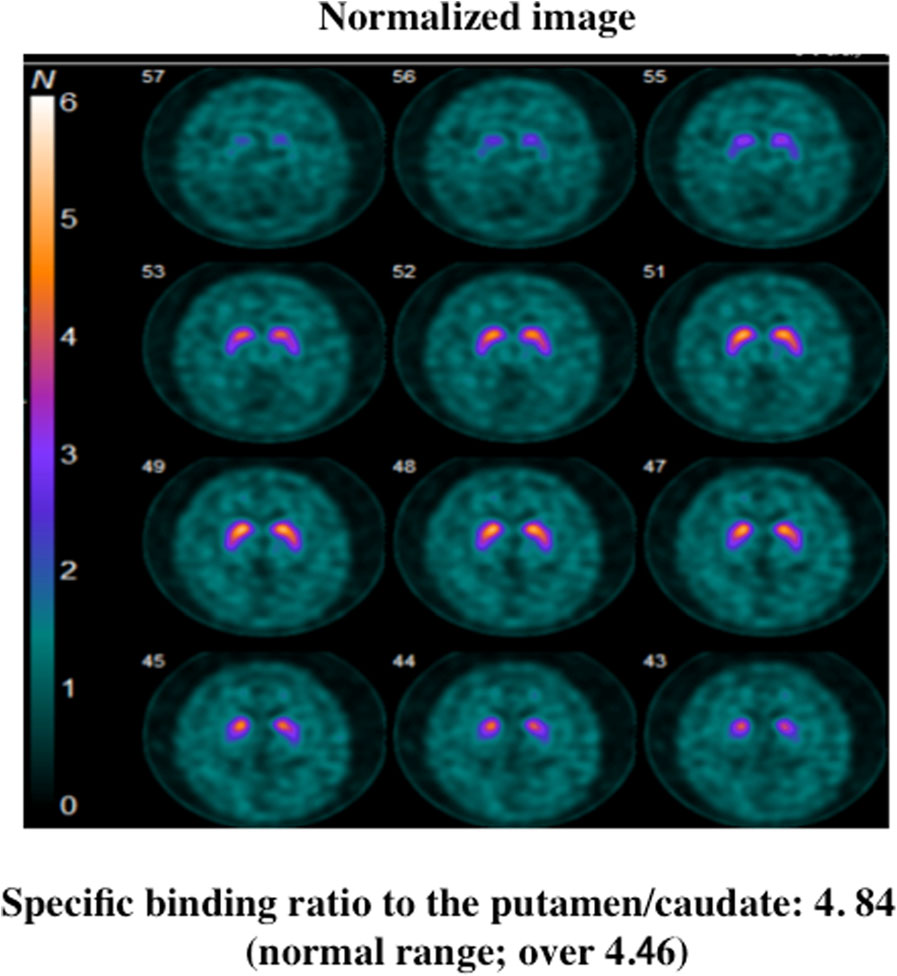
Dopamine transporter scintigraphy with ioflupane in this case. The significant reduced uptake was not confirmed in putamen or caudate nucleus

**Fig. 3:**
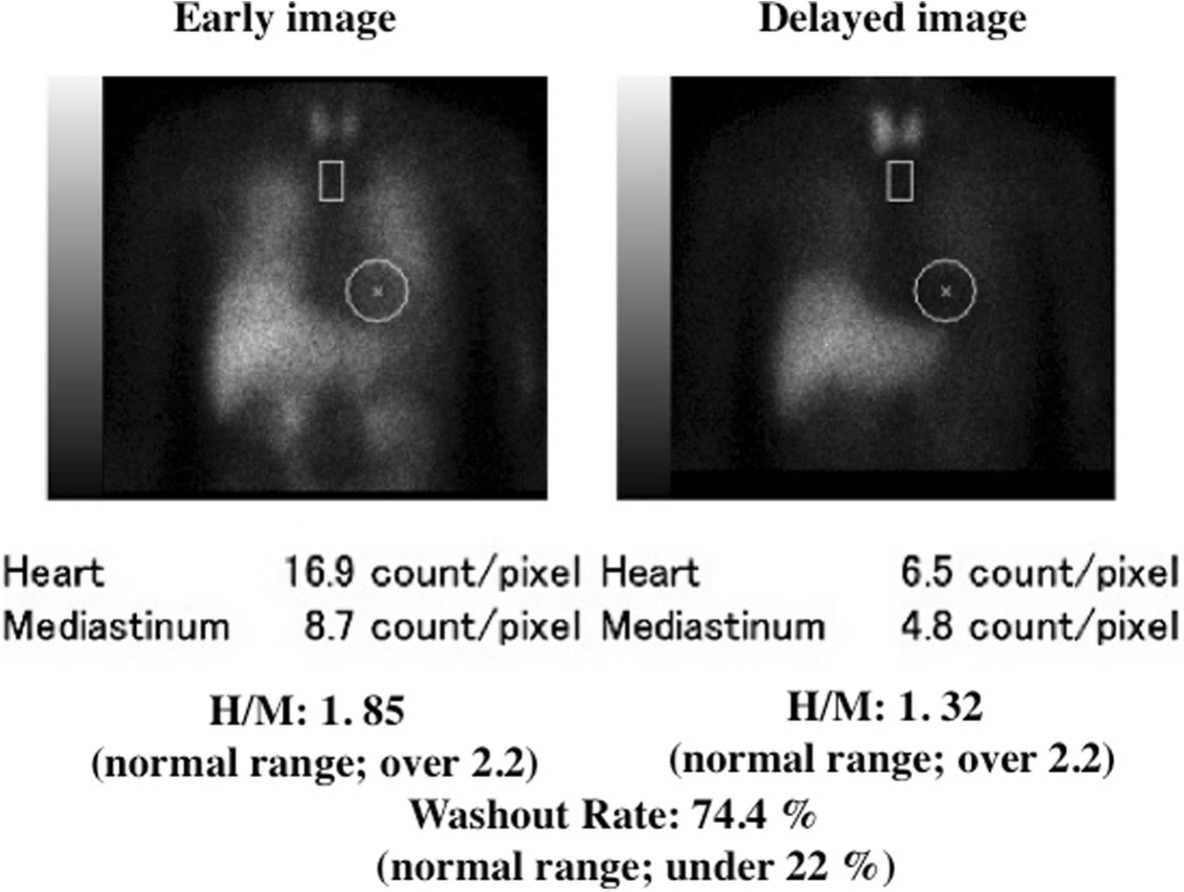
The anterior planar images (early and delayed) of ^123^I-MIBG cardiac scintigraphy in this case. Cardiac MIBG uptake and H/M ratio were reduced and washout rate was accelerated, compared to normal data. MIBG, meta-iodobenzylguanidine

From one year later, her husband noticed her talking and calling out in a loud voice during sleep once or twice a week, while falling out of bed occurred once or twice per month. Because these episodes gradually became more frequent at age 72, she underwent PSG during sleep for suspected RBD. Electroencephalography revealed no epileptic discharges. PSG study demonstrated that total sleep time (TST) was 433.5 min, REM sleep (%TST) was 10.7%, atonia index during REM sleep was 5.4 / hour, apnea-hypopnea index was 13.0 / hour (apnea index; 0.1 / hour), and periodic limb movements during sleep were 5.4 / hour. REM sleep without atonia was confirmed by PSG (Fig. 1). Evaluation of autonomic function showed loss of the sympathetic skin response related to sweating both at rest and with electrical stimulation, and a normal cutaneous vasomotor reflex response to electrical stimulation. Orthostatic hypotension was not evident, but she had occasional urinary incontinence. Dopamine transporter scintigraphy with ioflupane showed a normal finding that the mean specific binding ratio to the putamen/caudate was 4.84 (4.64 on the right side and 5.05 on the left side) (Fig. 2). Cardiac scintigraphy with iodine-123-labeled meta-iodobenzylguanidine (^123^I-MIBG) revealed a low heart/mediastinum ratio (early: 1.85; delayed: 1.32), indicating reduced uptake, and a high washout rate (74.4%) (Fig. 3).

## Discussion and conclusions

In this patient with SCA 31, recurrent episodes of talking and calling out loudly during sleep were noted six years after the onset of cerebellar ataxia and RBD was confirmed by detecting REM sleep without atonia on PSG. Previously, RBD has been reported in patients with various cerebellar degeneration syndromes [8, 10]. Multiple system atrophy is generally associated with accumulation of α-synuclein in the brain, and it has been reported that over 80% of these patients have RBD confirmed clinically or by PSG [7, 8]. Among the various subtypes of SCA, MJD is most frequently associated with RBD [11], but patients with other subtypes of SCA (such as SCA 2 or DRPLA) sometimes have symptoms suggestive of RBD or PSG confirmation of RBD [12, 13]. However, there has been no previous report of RBD in a patient with SCA 31 as far as we could determine.

Patients with α-synucleinopathies, including Parkinson’s disease and dementia with Lewy bodies, frequently show reduced uptake of MIBG on cardiac scintigraphy [14]. It has also been reported that patients with idiopathic RBD often have decreased MIBG uptake on cardiac scintigraphy [7, 8], and that many of them eventually develop an α-synucleinopathy such as Parkinson’s disease [9, 10]. Therefore, one reason why RBD occurred in our patient could be a possible association with α-synucleinopathy, since MIBG uptake was reduced on cardiac scintigraphy. However, involvement of the nigrostriatal dopaminergic network could not be confirmed by dopamine transporter scintigraphy.

Previous pathological studies and animal experiments have shown that complex impairment of several pontine nuclei (including the noradrenergic locus coeruleus and cholinergic nuclei), the substantia nigra, the hypothalamus, and the frontal cortex may be associated with the pathogenesis of RBD [7, 8]. Patients with MJD or SCA 2 often develop RBD, and brainstem dysfunction associated with progression of the pathologic process of SCA may lead to the onset of RBD [11, 12]. It is possible that RBD occurred in our patient due to pathologic changes associated with SCA 31. While pathological investigation of the brainstem has only revealed involvement of the inferior olivary nucleus in SCA 31 [15], it is still possible that sleep disorders like RBD arise from brainstem involvement, because pyramidal tract symptoms and involuntary movements suggesting brainstem lesions have been reported in SCA 31 patients [2]. Clinical detection of extrapyramidal symptoms or cognitive impairment would provide more information about the pathophysiological mechanism in the present case.

In our patient, cardiac scintigraphy revealed a decrease of MIBG uptake. As well as in patients with α-synucleinopathies, this abnormality is often found in patients with diabetic autonomic neuropathy or familial amyloidotic polyneuropathy [16]. Thus, there may be subclinical autonomic involvement in SCA 31, because urinary incontinence and a reduced sweating response were noted in our patient and neurogenic bladder was reported in another patient with this disease [17]. There is the limitation in this study due to a single case report and since the possibility of the future complication of α-synucleinopathy can not be denied, though current cognitive impairments or parkinsonian symptoms were not found and dopamine transporter scintigraphy with ioflupane showed a normal finding. In the future, evaluation of autonomic function, assessment of the frequency of RBD, and performance of cardiac MIBG scintigraphy in a larger number of SCA 31 patients could be useful to resolve important issues regarding the mechanism of RBD.

## Abbreviations

^123^I-MIBG: Iodine-123-labeled meta-iodobenzylguanidine
PSG: Polysomnography
RBD: REM sleep behavior disorder
REM: Rapid eye movement
SCA: Spinocerebellar ataxia
